# Auto-Craniotomy With Leaves Implantation on the Surface of the Brain Following a Road Traffic Accident

**DOI:** 10.7759/cureus.31631

**Published:** 2022-11-18

**Authors:** Rajiv Neupane, Dipak K Yadav, Samiksha Raut, Prakash Kafle

**Affiliations:** 1 Department of Neurosurgery, Nobel Medical College Teaching Hospitals, Biratnagar, NPL; 2 Department of General Surgery, Nobel Medical College Teaching Hosptials, Biratnagar, NPL

**Keywords:** head injury, leaf, road traffic accident, foreign body, auto-craniotomy

## Abstract

The development of modern vehicles that are not compatible with the roads, the rising burden of vehicles on narrow roads, and the recklessness of the vehicle's driver and pedestrians have resulted in an increase in uncommon and severe forms of head injury. We present a case of non-fatal autocraniotomy with leaf implantation inside the cranium. Foreign body implantation merit certain management adaptation from other traumatic head injuries. This case highlights the detachment of the frontal bone of the skull on the left side with a Superior Sagittal Sinus tear and leaf implanted inside the cranium. Early assessment of the patient with computed tomography (CT) of the head to identify surgically removable foreign bodies and thorough washing of the brain and scalp is recommended.

## Introduction

Worldwide road traffic injuries (RTI) account for 2.1% of global mortality. Out of the total global mortality due to RTI, developing nations contribute to 85% of the deaths [1]. As per the data published by the World Health Organization in 2018, RTI led to 3.07% of the total death rate in Nepal [2]. A study in eastern Nepal by Kafle documented road traffic accidents as the most common cause (76%) of traumatic brain injury, with an overall mortality rate of 11.17% [3]. Most RTIs occur in hilly districts due to difficult terrain, and there is a need for thorough investigations of the major causes of RTIs [4]. In neurosurgery, a craniotomy is needed to expose the required part of the brain after removing part of the bone of the skull. The removed bone is replaced after the surgery [5]. This case presents insight into autocraniotomy following RTI and implantation of the leaf as a foreign body on the brain surface.

## Case presentation

A 39-year-old married male had RTI after slipping off a motorcycle while riding without a helmet on a steep road. The patient had a loss of consciousness and was brought to us via ambulance. The scalp was covered and pressed with a towel that was fully soaked in blood. There were multiple abrasions and lacerations on the right forearm and right arm.

In the emergency department, the spine was stabilized with a hard cervical collar and was resuscitated. The patient was unconscious with no eye-opening (E1), no verbal response (V1), and a localizing response to pain (M5). Pupils were bilaterally equal and reactive to light. There was no history of vomiting or bleeding from the ear, nose, or throat. There was no nasal or ear discharge.

**Figure 1:**
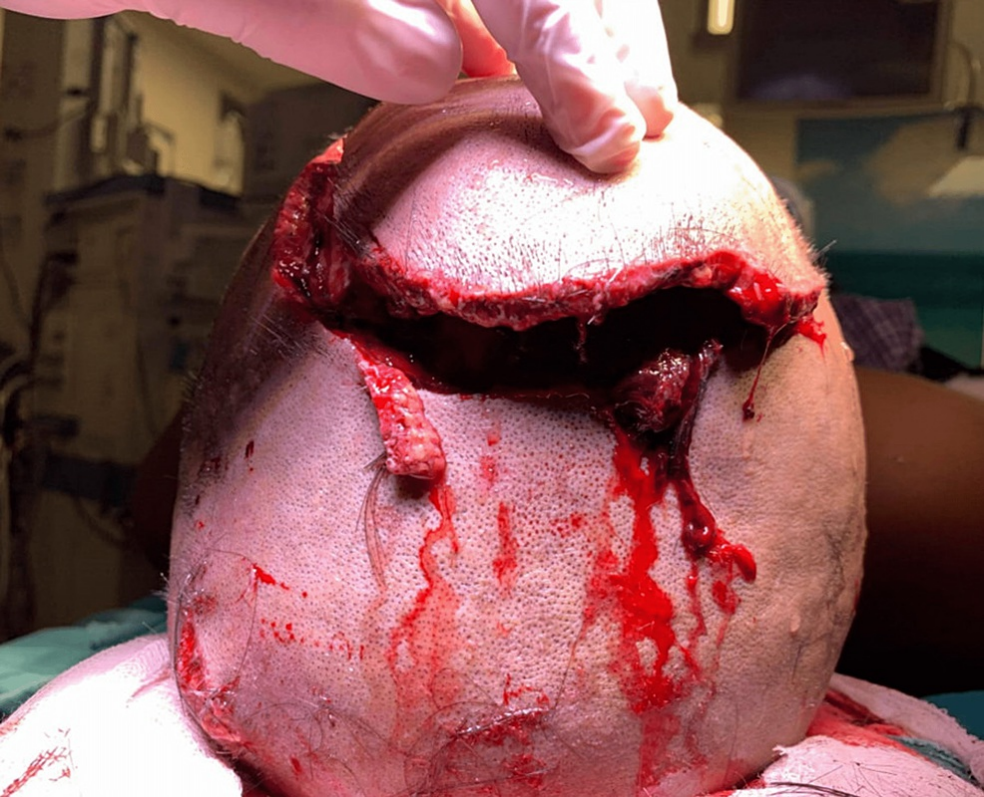
De-gloving scalp injury with bilateral frontal bone fracture

Local examination revealed a de-gloving scalp laceration (Figure 1) with a bilateral frontal bone fracture traversing the superior sagittal sinus. The fracture extended from just above the coronal suture up to the bilateral supraorbital ridge. The wound was around 10 cm × 8 cm in size. Active bleeding was present around the area of the superior sagittal sinus.

**Figure 2:**
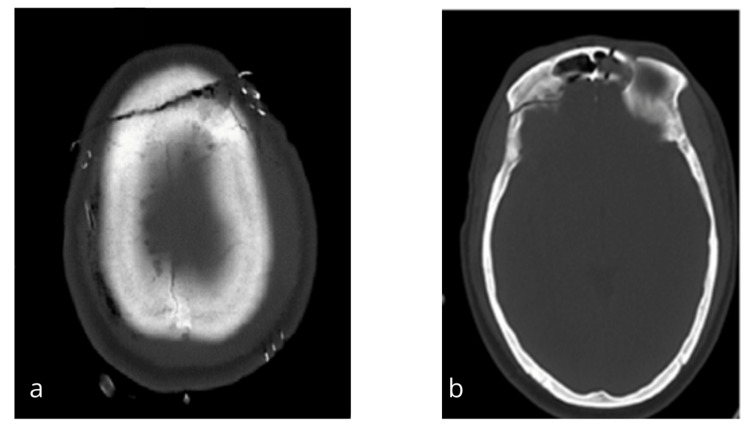
(a) Preoperative CT head (bone window) showing segmented frontal bone. (b) Preoperative CT head showing the fracture line extending to the frontal sinus.

A computed tomography (CT) scan of the head (Figure 2) demonstrated the frontal autocraniotomy involving the superior sagittal sinus and frontal air sinus. There was a contusion on the right frontal lobe as well. There was an associated extradural hematoma over the frontal region but no mass effect. CT cervical spine screening revealed no abnormalities.

**Figure 3:**
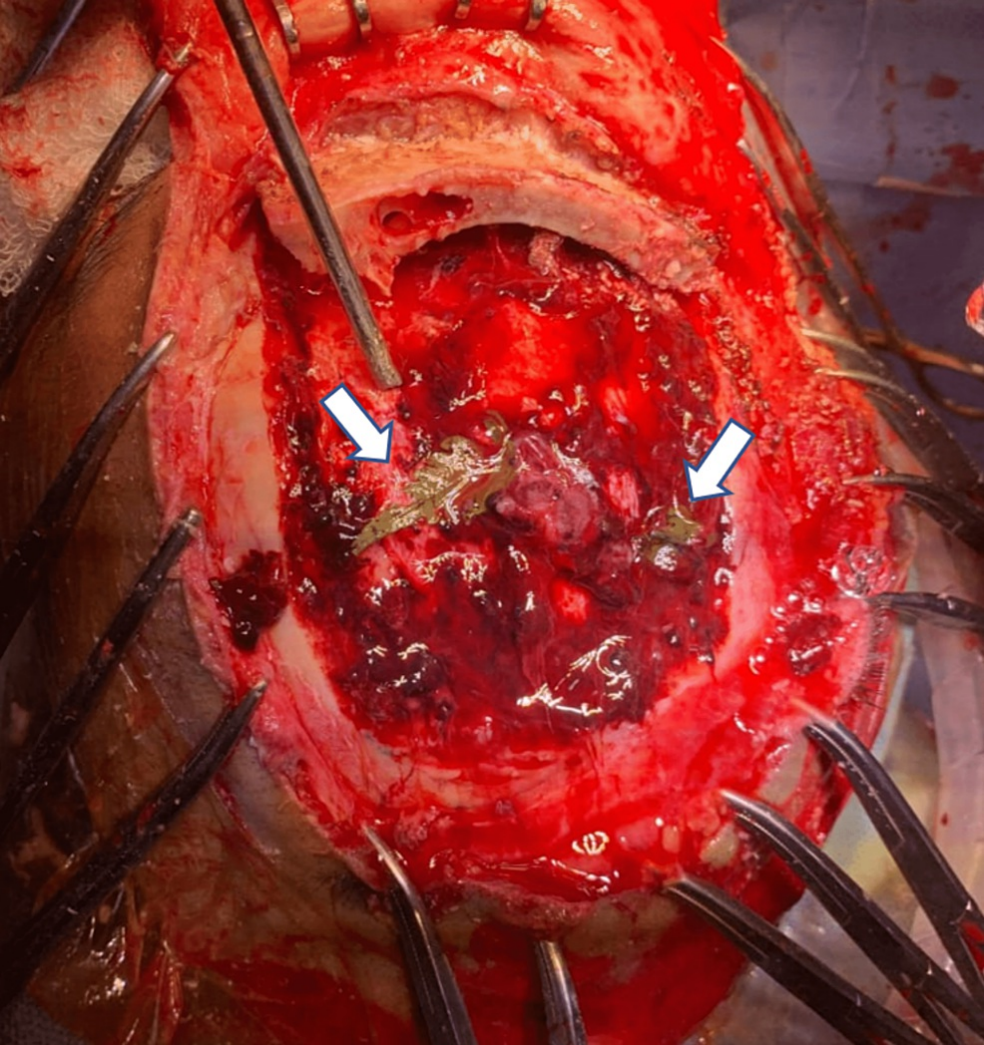
Green leaves as foreign body on the surface of brain (white arrow).

The patient was started on prophylactic antibiotics and anticonvulsants. A thorough cleaning of the wound was done in the operating room. A lacerated wound on the left side of the scalp was extended, and an auto-craniotomized bone fragment was removed. On removal of the bone fragment, foreign bodies were visualized, which appeared to be green leaves (Figure 3). There were two of them, with the largest measuring about 4 cm × 2 cm in size.

Thorough irrigation with hydrogen peroxide (H_2_O_2_) at 3% concentration and normal saline was done. The superior sagittal sinus tear was repaired and packed with Surgicel and Abgel (Ethicon, Somerville, NJ). The frontal air sinus was also packed with Abgel and bone wax. Hemostasis was obtained, and the bone was then fixed with a miniplate and screws. A sub-galeal drain was kept, and the scalp was closed in layers. The patient was continued on antibiotics and antiepileptic medications post-operatively. A secondary survey revealed both bone fractures of the right forearm, which were initially managed conservatively with the orthopedic team in the neuro-intensive care unit (ICU). The patient spent a total of three days in the ICU and was later shifted to the neurosurgery ward. His total of seven days of the postoperative period were uneventful, and he never had an episode of abnormal body movement. His Glasgow Outcome Score (GOS) was 5 out of 5 on postoperative day 7. Following full improvement in neurological status and a review of the CT scan, the patient was shifted to the orthopedic unit for open reduction and internal fixation of both bone fractures (right forearm) with plates and screws.

## Discussion

This is probably the first reported case of green leaves as a foreign body implanted inside the cranium following auto-craniotomy. The cranium vault over the frontal region got craniotomized itself due to a high-velocity injury during a road traffic accident. Along with this, there was a superior sagittal sinus tear and a frontal air sinus wall fracture. Several penetrating objects, including blades, nails, pencils, splinters of wood, and wire, have been reported inside the skull and brain [6]. In a study [7], a fracture of the skull was seen in almost 80% of patients with a history of road traffic accidents. In the same study, a temporal bone fracture was the most common (58.6%). In a systematic review [4], a few possible causes of accidents mentioned are alcohol consumption, pedestrian road behavior, and inappropriate driving. A cohort study concluded that patients who had a traumatic brain injury and survived the initial acute phase of care can have long-term morbidity in comparison to the general population, despite the initial level of injury severity [8].

## Conclusions

A craniotomy is a common procedure in neurosurgery in which a part of the skull bone is removed temporarily to access the brain for different procedures. Auto-craniotomy is the complete chopping off of a portion of the skull bone on its own in a similar fashion to craniotomy done in neurosurgery, either by fall, trauma, or high-velocity injuries like RTA. The implantation of leaves along with auto-craniotomy following a road traffic accident is a rare incident encountered. The cranium, which is a "closed and tough box" surrounded by a bony structure, can be cleaved off following a high-velocity RTA, and preservation of the bony fragment is crucial for good repair, postoperative outcome, and survival. The repair of the dural sinuses, which are torn during auto-craniotomy, is crucial to securing a good repair for controlling the bleeding and preventing extra-dural collections during the postoperative period. Cleaning and removal of foreign bodies, as well as appropriate postoperative antibiotic coverage, also form important steps in the operative approach, as with the other contaminated wounds, to prevent postoperative complications like meningitis and brain abscess, as well as early good recovery and overall prognosis.
